# Deciding Whether to Include Non‐Randomized Studies of Interventions in Systematic Reviews: A Tutorial

**DOI:** 10.1002/cesm.70109

**Published:** 2026-11-06

**Authors:** Nicola Lindson, Hugh Sharma Waddington, Ella Flemyng, Peter Tugwell, David B. Wilson, Jamie Hartmann‐Boyce

**Affiliations:** ^1^ Nuffield Department of Primary Care Health Sciences University of Oxford Oxford UK; ^2^ Department of Population Health London School of Hygiene & Tropical Medicine London UK; ^3^ Cochrane Central Executive Cochrane London UK; ^4^ Department of Medicine University of Ottawa Ottawa Canada; ^5^ Bruyère Health Research Institute Ottawa Canada; ^6^ Ottawa Hospital Research Institute Ottawa Canada; ^7^ School of Epidemiology and Public Health University of Ottawa Ottawa Canada; ^8^ Department of Criminology, Law and Society George Mason University George Mason Virginia USA; ^9^ Department of Health Promotion and Policy University of Massachusetts Amherst Massachusetts USA

**Keywords:** medicine, medline, psychological intervention, systematic review

## Abstract

This tutorial provides practical guidance on deciding whether to include non‐randomized studies of interventions (NRSI) in systematic reviews. Drawing on existing Cochrane and GRADE guidance, it outlines circumstances in which NRSI may provide valuable evidence alongside or in the absence of randomized controlled trials. A practical checklist supports review authors in making decisions about study eligibility for each PICO at the protocol stage, considering the availability and limitations of randomized evidence, the outcomes of interest, equity, and generalizability.

## Graphical abstract

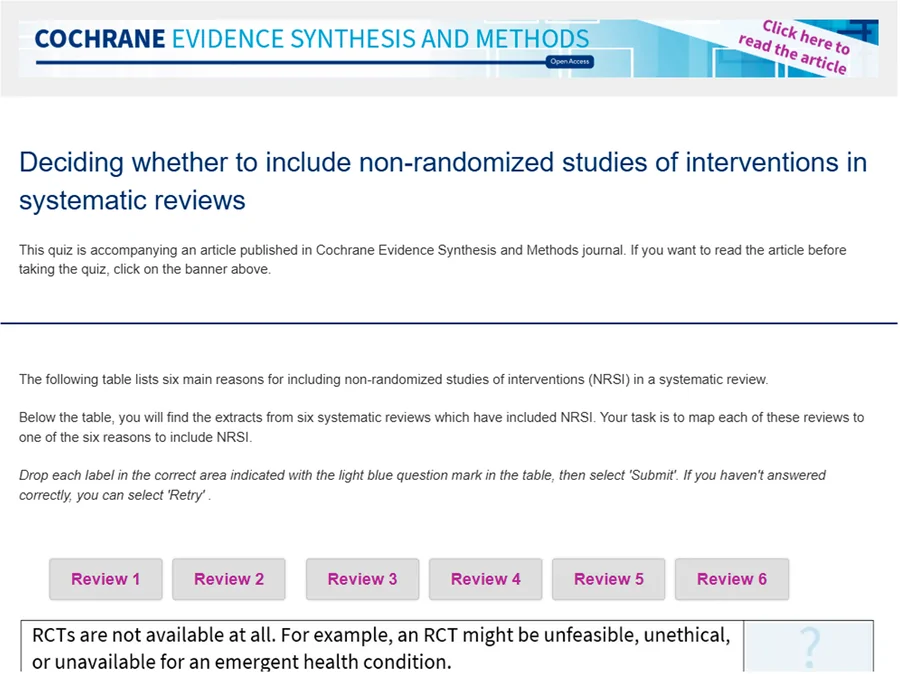

## 1 What Are NRSI?

NRSIs are defined in the [1] Cochrane Handbook for Systematic Reviews of Interventions v6.5 as any quantitative study estimating the effects (benefit or harm) of an intervention that does not use randomization to allocate individuals (or groups of individuals) to intervention groups. NRSI use a variety of designs and methods to estimate and quantify the causal effect on the outcome(s) of interest of a healthcare intervention, program, or policy (In the social sciences, the term “quasi‐experimental design” [QED] is used to describe non‐randomized studies of intervention effects [2]. NRSI, where the researchers have no role in treatment allocation and which are frequently conducted using existing data, are also called “natural experiments” [3]). In an NRSI, the healthcare intervention may be assigned according to a known rule, such as a clinician who prescribes treatment based on a pre‐test measure (e.g., blood pressure), or according to other (often unknown) non‐randomized decision‐making rules by planners, clinicians, or patients themselves [4].

NRSIs are increasingly recognized to have an important role to play in systematic reviews of healthcare interventions [5]. For example, they may be particularly useful in answering specific synthesis questions within a systematic review, for example, questions about long‐term, rare, and/or adverse events (harms), or to address important equity considerations such as effects for specific populations. NRSI may also be important in syntheses that examine the effects of interventions, programs, and policies delivered under real‐world settings, using routine methods of assignment of patients or other groups to a healthcare intervention [6].

### 1.1 What Do I Need to Do Before Considering Whether to Include NRSI?

Before deciding whether to include NRSI, you should have defined your systematic review aim, articulated the review populations, interventions, comparators and outcomes (PICO) criteria, together with the PICO for each synthesis (e.g., for a particular population, intervention, or outcome) addressed in the systematic review (see Box 1). You should also have conducted a scoping exercise of the randomized controlled trial (RCT) and NRSI evidence, and/or have sufficient knowledge of the subject area to know whether there are relevant randomized controlled trials.

Box 1 **PICO for each synthesis addressed in the systematic review.**

PICO is an acronym used to help shape clinical questions and inclusion criteria for systematic reviews of interventions. It stands for:

- Populations: the individuals or groups of interest.
- Interventions: the interventions, programs, or policies of interest.
- Comparators: the conditions to which the interventions will be compared.
- Outcomes: the variables that assess the effects.

Systematic reviews usually include multiple PICOs. For example, important outcomes may include both intended and unintended consequences (including adverse events), be short term or measured over a long period, sometimes many years later; the population may include all eligible individuals, but specific sub‐questions may relate to the most vulnerable groups such as those with comorbid health issues; the intervention may have been investigated in explanatory (proof‐of‐concept) and routine care settings; the comparator may include those receiving standard care (practice as usual), a wait‐list, an alternate intervention, or nothing at all.

The purpose of this document is to help Cochrane authors define eligible study designs for each PICO at the protocol stage of their review. See also [1] Chapter 2 of the Cochrane Handbook for Systematic Reviews of Interventions.

### 1.2 When Should I Include NRSI in My Intervention Review?

RCTs, when designed and conducted rigorously, are widely considered to be the least biased form of primary evidence when assessing the effects of healthcare interventions [1, 7]. Therefore, a Cochrane systematic review of the effects of a healthcare intervention should always include relevant RCTs if available. However, NRSI may also be an acceptable source of evidence in the following circumstances:

1. For evidence about unintended consequences or adverse events (harms), which RCTs may not measure, or only measure imprecisely (i.e., they are underpowered). NRSI may also be at lower risk of bias in measuring unintended consequences than intended consequences, in some circumstances, because initial allocation to treatment conditions by practitioners or researchers is less likely to be associated with the probability of an outcome that is not anticipated [8]. An example is a review of the effects of infant sleeping position on mortality [9].
2. If the PICO relates to the effects of interventions conducted in real‐world settings rather than under highly controlled settings, especially in settings or fields where the conduct of an RCT would be challenging or uncommon; for example, when estimating the effects of juvenile curfews on offending [10].
3. For evidence about long‐term effects, where RCTs may be impractical, for example, due to the need to isolate control groups from the healthcare intervention over long periods. To take one example, a review of the effects of mass deworming interventions incorporated NRSI to measure long‐term effects on education and work [11].
4. For outcomes measured among groups of participants who are not well represented in other studies, such as those included under PROGRESS‐Plus [12] (PROGRESS stands for place of residence, race/ethnicity/culture/language, occupation, gender/sex, religion, education, socioeconomic status, and social capital; Plus refers to personal characteristics associated with discrimination [e.g., disability], features of relationships [e.g., parents who smoked], and instances when a person is temporarily disadvantaged [e.g., respite care]). For example, a review of the effects of farmer field schools incorporated NRSI undertaken among women farmers and hard‐to‐reach groups [13].
5. For rare primary outcomes, which are not necessarily unintended or adverse, for which evidence from RCTs may be underpowered. For example, a review of the effects of water, sanitation, and hygiene interventions on mortality in childhood incorporated both RCTs and NRSI [14].
6. For emergent health conditions, if RCTs are likely to take longer to design and conduct than the decision‐making cycle requires; for example, a review on the effects of personal protective equipment in reducing infection conducted in response to the COVID‐19 pandemic [15].

Some publications reporting NRSI may also be known and used in policy and practice but be ineligible for a systematic review if it only plans to include RCTs. In these cases, reviewing this evidence alongside other systematically identified eligible studies can help raise awareness among decision makers about any concerns regarding these studies (drawing on appropriate risk of bias assessment) (https://www.cochrane.org/learn/courses-and-resources/cochrane-methodology/risk-bias/robins-i-tool-resources-and-reporting-guidance).

The exercise below (Box 2) can help you decide whether to include NRSI in your review of effects of a healthcare intervention. Your initial scoping of the literature should provide an indication of the evidence available for each review PICO/synthesis and facilitate this.

Box 2 **Should you consider including evidence from NRSI in your systematic review of effects? This exercise should be considered for each proposed PICO in your systematic review protocol [1] adapted from Section 24.1.1. of the Cochrane Handbook for Systematic Reviews of Interventions and using Schünemann et al. [16] and Cuello‐Garcia et al. [17].**

**Consider including NRSI evidence if (tick all that apply):**

RCTs are not available at all. For example, an RCT might be unfeasible, unethical, or unavailable for an emergent health condition.^a^

RCT evidence is available but judged to be sparse (of limited quantity), uncertain (imprecise), or seriously indirect (meaning the evidence does not map directly onto your PICO).^a^

PICO outcomes concern unintended or adverse events (harms).^b^

PICO outcomes are likely only to occur in the long‐term and/or are rare.^b^

Inclusion may facilitate investigation of important equity or generalizability considerations that are considered as high priorities for the review. For example, if RCTs have only been conducted in high‐income contexts or socioeconomically advantaged groups, or another relevant characteristic, where there is reason to think that there may be differences in what the evidence shows in these different groups.^c^

Evidence from NRSI has been used for clinical or policy decision‐making in your area of interest. In this case, end users of your review may benefit from you formally assessing this evidence.

***→If you have ticked one or more of these statements, then you should consider including NRSI.***

Other reasons to consider including NRSI are:

- Effects differ between NRSI and RCT evidence in your area of interest, and you want to explore this further.
- It will enhance the ability of your review to address questions about moderator effects (e.g., in meta‐regression analysis).
- It can inform your baseline absolute risk figures used in summary of findings tables, to add context in your introduction or discussion sections, or to investigate correlation between surrogate outcomes and patient‐important outcomes [18].

***Notes***: (a) GRADE refers to this as “replacement evidence”; (b) GRADE refers to this as “sequential evidence”; (c) GRADE refers to this as “complementary evidence.” NRSI may provide less certain evidence than well‐conducted RCTs so including them does not necessarily result in higher quality or more consistent findings. For Cochrane reviews, it is advised that NRSIs at critical risk of bias are not included in the synthesis and therefore do not contribute to your review findings.

**Abbreviations**: NRSI, non‐randomized studies of interventions; PICO, Population, Intervention, Comparator, Outcome; RCT, randomized controlled trial.

### 1.3 So, You've Decided to Include NRSI in Your Review—What Next?

This tutorial focuses on whether to include NRSI in your review. If you decide to do so, substantial further methodological work is needed, which is outside the scope of this tutorial. If you do decide to include NRSI, your author team should include people with expert knowledge of NRSI methods.

Inclusion of NRSI in systematic reviews is an area where methods are evolving quickly; hence, authors should refer to relevant methods guidance, including [1] Chapter 24 of the *Cochrane Handbook for Systematic Reviews of Interventions*, which authors are advised to consider and should adhere to in their systematic review. Hartmann‐Boyce et al. [4] contains further guidance on how to distinguish between different types of NRSI, as relates to assumptions required for causal inference (out of scope for this tutorial). The Grading of Recommendation, Assessment, Development and Evaluation (GRADE) Working Group has also developed useful guidance on incorporating NRSI in systematic reviews [16, 17].

### 1.4 Further Reading

This tutorial is accompanied by a Cochrane micro‐learning module, available at the following link: https://share.gomolearning.com/sharelink/d479fbe4da82699216a98464659cb8d90721d6bbaab9cd7ded/.

Another linked paper that resulted from the same project is available at the following link: https://ebm.bmj.com/content/early/2026/01/06/bmjebm-2025-114125.

## Author Contributions

**Nicola Lindson:** conceptualization, funding acquisition, writing – original draft, project administration, investigation. **Hugh Sharma Waddington:** writing – original draft, writing – review and editing, investigation. **Ella Flemyng:** conceptualization, funding acquisition, investigation, writing – review and editing. **Peter Tugwell:** writing – review and editing, supervision. **David B. Wilson:** writing – review and editing, supervision. **Jamie Hartmann‐Boyce:** conceptualization, investigation, funding acquisition, project administration.

## Ethics Statement

These aspects are not relevant to this submission, as the work is not classified as research, and thus ethics approval and patient consent was not required. Existing guidance, for example, from the Cochrane Handbook for Systematic Reviews of Interventions v6.5 [1] and GRADE [16, 17], sets out a range of considerations regarding inclusion of non‐randomized studies of interventions (NRSI) in systematic reviews on the effects of healthcare interventions. This tutorial summarizes existing guidance and aims to help you think through whether NRSI should be included in your systematic review.

## Conflicts of Interest

N.L. was employed by Oxford University Hospitals Trust between 2018 and 2023 to provide teaching on Cochrane Review methods on behalf of Cochrane UK. E.F. is employed by the Cochrane Collaboration and completed this work as part of that employment. They are also on the Editorial Board of Cochrane Evidence Synthesis and Methods, but was not involved in any editorial processing or decisions related to this manuscript. P.T. is co‐convenor of the Cochrane Equity Methods Group and the Cochrane Health Equity Thematic Group. They also receive a yearly honorarium for sitting on the advisory committee of the Reformulary Group and are the unpaid Chair of the Management Group for a registered non‐profit independent medical research organization‐OMERACT. The latter receive funding from AbbVie, AstraZeneca, Aurinia, BMS, Centrexion, GSK, Horizon Pharma Inc, Janssen, Novartis, Pfizer, and Sparrow. J.H.‐B. is a Cochrane Senior Editor, but was not involved in any editorial processing or decisions related to this manuscript. The other authors declare no conflicts of interest.

## Data Availability

Data sharing is not applicable to this article as no data sets were generated or analyzed during the current study.
